# Designing a Prognostic Scoring System for Predicting the Outcomes of Proximal Fifth Metatarsal Fractures at 20 Weeks

**Published:** 2015-03

**Authors:** Mohammad Ali Tahririan, Amir Momeni, Amir Moayednia, Elham Yousefi

**Affiliations:** 1 Department of Orthopaedic Surgery, Kashani University Hospital, Isfahan University of Medical Sciences, Isfahan, Iran;; 2 Department of Orthopaedic Surgery, Isfahan University of Medical Sciences, Isfahan, Iran;; 3 Department of Physical Medicine and Rehabilitation, Isfahan University of Medical Sciences, Isfahan, Iran

**Keywords:** Metatarsal bone, Fracture, Prognosis

## Abstract

**Background:**

Fractures of the proximal fifth metatarsal bone are among the most common fractures observed in the foot and their classification and management has been subject to much discussion and disagreement. In this study, we aim to identify and quantify the effect of possible predictors of the outcome of the treatment of proximal fifth metatarsal fractures.

**Methods:**

Patients with established proximal fifth metatarsal fractures were enrolled in this prospective cohort and the outcome of their treatment was assessed using the AOFAS mid foot scale at 6 and 20 weeks.

**Results:**

143 patients were included in the study. Our study showed that displacement, weight and type III fractures were significant independent predictors of poor outcome at 6 weeks while at 20 weeks in addition to these factors, gender and diabetes mellitus were also shown to be significant independent predictors of poor outcome. A scoring system was designed by assigning weight to these factors and it was shown to be a strong predictor of outcome at 20 weeks.

**Conclusion:**

We recommend that our scoring system would help surgeons to decide whether patients’ prognostic factors are significant enough for him/her to opt for a surgical approach to treatment rather than a conservative approach

## Introduction

Fractures of the proximal fifth metatarsal bone are among the most common fractures observed in the foot.^1^^,^^2^ The proximal fifth metatarsal bone has been considered to have three anatomical zones: the styloid process (zone I), meta-diaphyseal area (zone II) and proximal diaphysis (zone III).^3^ Evidence has shown that zone I is most commonly involved in fracture of the base of the fifth metatarsal bone.^3^

Classically, fractures of the proximal fifth metatarsal (Dameron zone I) have been classified into three types: type I fractures involve the tip of the tuberosity, type II fractures are oblique fractures starting from the base of the metatarsal running into the metatarsocuboid joint and type III fractures are transverse fractures that involve fourth metatarsal articulation.^3^ This classification is meant to be predictive of the outcome where type I fractures and tuberosity avulsions are believed to carry a better prognosis and a higher chance for complete healing than the more distal fractures.^4^^,^^5^

Type I fractures have been associated with sports-related injuries in males while type II and type III fractures are usually caused by inversion injuries.^3^^,^^6^ Factors such as increased metatarsus adductus angle have been shown to increase the vulnerability of individuals to Jones fractures^7^ while other factors have been associated with poor response to treatment. In general, Jones fractures are considered prone to prolonged healing time and non-union.^8^

Type I fractures are usually treated conservatively with a non-weight bearing short leg cast for 6 to 8 weeks, type II fractures are treated either conservatively or surgically but type III fractures especially if there is considerable displacement are usually treated surgically.^9^ In athletes and active adults, the current evidence recommends that type II and type III be treated surgically with the use of intramedullary screw fixation.^10^ In patients with lesser injuries and minimal displacement there have been arguments for the use of functional treatment - in the form of elasticated support bandage - in lieu of casts, with some studies reporting improved outcomes.^11^

The choice of treatment for proximal fifth metatarsal fractures is controversial; the current approach as supported by the best evidence available, recommends surgery in patients who have fractures with considerable displacement (more than 2 mm) or in patients with extensive involvement of the cubometatarsal joint (more than 30%)^8^ with more conservative approaches considered for lesser injuries. The choice of treatment depends on radiological and clinical findings among with the judgement of the orthopaedic surgeon.

A need for identifying predictors that can determine the outcome of these fractures exists; such predictors can guide the choice of treatment and be a support tool that facilitates the decision making process of the orthopaedic surgeon. In this study, we aim to identify and quantify the effect of possible predictors on the outcome of the treatment of proximal fifth metatarsal fractures. 

## Methods and Materials

**Figure 1:**
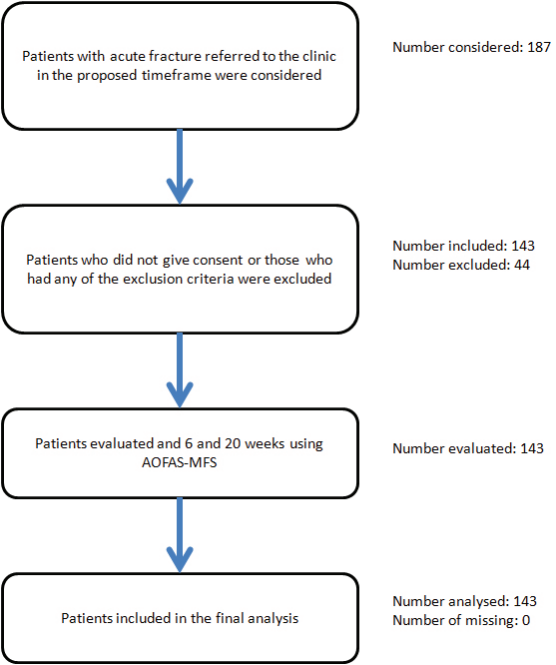
Study flowchart

This prospective cohort was undertaken in the orthopaedic clinic of Kashani hospital in Isfahan (Iran) during two years from April 2011 until April 2013. Patient selection is shown in figure 1. The aim of the study was to identify potential predictors that could affect the outcome of the fracture in our patients.

Patients with acute (less than 5 days) foot complaints or foot trauma that were referred to our clinic were evaluated clinically by an orthopaedist and by X-ray. Anyone with a confirmed (radiologically proven) fracture of the proximal fifth metatarsal bone - who did not meet any of the exclusion criteria - was included in the study after obtaining informed consent. The exclusion criteria were previous fifth metatarsal fracture, need for surgical intervention, multiple fractures, previous foot deformity, fractures involving the diaphysis of the bone, open fractures and chronic foot complaints.

The patients were evaluated after obtaining informed consent and their condition including the severity and type of the fracture as well as the degree of displacement was documented. All patients were treated by short leg casts for six weeks with weight bearing after the second week. They were evaluated by an orthopaedist at six and twenty weeks. The evaluation included a demographic and clinical questionnaire regarding the general health of the participants as well as the AOFAS mid foot scale (MFS); this scale is a standardized tool used for the evaluation of mid foot complaints (including proximal fifth metatarsal fractures) (table 1). This tool assigns scores to patients based on three categories: pain (40 points), function (45 points) and alignment (15 points), with a maximum possible score of 100 (limited or no complaint) and a minimum possible score of zero (complete or major disability).^12^ AOFAS scale has been shown to have acceptable validity and reliability in assessing mid foot problems.^13^ The pain is assessed by asking the patient about the degree and frequency of pain in his/her mid foot while function and alignment are evaluated by a trained orthopaedist. The goal of the treatment is to maximize patient’s MFS. The data obtained was entered into a database and analyzed using IBM SPSS (version 18).

**Table 1:** AOFAS midfoot scale

**Midfoot Scale (100 Points Total)**	
Pain (40 points)	
None	40
Mild, occasional	30
Moderate, daily	20
Severe, almost always present	0
Function (45 points)	
Activity limitations, support	
No limitations, no support	10
No limitation of daily activities, limitation of recreational activities, no support	7
Limited daily and recreational activities, cane	4
Severe limitation of daily and recreational activities, walker, crutches, wheelchair	0
Footwear requirements	
Fashionable, conventional shoes, no insert required	5
Comfort footwear, shoe insert	3
Modified shoes or brace	0
Maximum walking distance, blocks	
Greater than 6	10
4-6	7
1-3	4
Less than 1	0
Walking surfaces	
No difficulty on any surface	10
Some difficulty on uneven terrain, stairs, inclines, ladders	5
Severe difficulty on uneven terrain, stairs, inclines, ladders	0
Gait abnormality	
None, slight	10
Obvious	5
Marked	0
Alignment (15 points)	
Good, plantigrade foot, midfoot well aligned	15
Fair, plantigrade foot, some degree of midfoot malalignment observed, no symptoms	8
Poor, nonplantigrade foot, severe malalignment, symptoms	0

Descriptive analysis was used to show the frequencies and characteristics of the predictors. These predictors were assessed in a univariate analysis for their correlation to outcome (as measured by MFS at 6 and 20 weeks). A multivariate analysis (multivariate linear regression; Enter method) was also performed in order to find independent predictors that could affect the outcome at 6 and 20 weeks. Finally, predictors that were shown to be independently and significantly correlated to the outcome at 20 weeks were used to design a scoring system that could predict possible unfavourable outcome in patients with proximal fifth metatarsal fractures treated conservatively with a short leg cast. The outcome (MFS score at 20 weeks) was dichotomized by considering patient with a higher than mean score as having a favourable outcome and those with a lower than mean score as having a poor outcome. Using this dichotomized variable and ROC curve provided the best possible cut-off point for our scoring system.

The scoring system was internally validated by using the bootstrap method and the power of the scoring system in predicting the outcome was assessed in 1000 iterations of random sampling from our data.

This study was approved by the Ethics Committee of Isfahan University of Medical Sciences. 

## Results

*Clinical Characteristics*

143 patients with mid foot fracture were enrolled in the study and were revisited in 6 and 20 weeks to assess their condition; all patients finished the study and completed the follow up. The mean age of the participants was 40.06 years (95% CI: 37.93-42.19). 100 patients were male (69.9%) and 43 were female (30.1%). 44 patients had styloid avulsion fractures with involvement of the metatarsocuboid joint (type I, 30.8%), 71 patients had involvement of the fourth and fifth metatarsal joint (type II, 49.7%) and finally 28 patients had proximal fracture of the metaphysis of the fourth and fifth metatarsal bones (type III, 19.6%). 19 patients had fractures with a displacement of greater than 2 mm (13.3%). The average weight of the participants was 80.47 kg (95% CI: 79.27-81.67). The average height of the participants was 1.72 m (95% CI: 1.71-1.72). The average BMI of the patients was 27.21 (95% CI: 26.79-27.63). 6 patients had diabetes mellitus (4.2%) and 43 were smokers (30.1%). 

The average AOFAS mid foot scale at 6 weeks and 20 weeks was 77.71 (95% CI: 76.79-78.63) and 92.77 (95% CI: 91.79-93.75) respectively.

*Univariate and Multivariate Analysis*

By comparing the predictive factors with the mid foot scale scores at 6 weeks, the following factors were found to be significantly associated with a poor outcome: displacement (P<0.001), weight (P=0.002), BMI (P=0.004), and type III fracture (P<0.001). Type I and type II fractures were associated with a better outcome (P=0.004 and 0.022 respectively). 

The comparison of the predictive factors with mid foot scale scores at 20 weeks identified the following factors as having a negative impact on the outcome: displacement (P=0.001), weight (P<0.001), BMI (P<0.001), and type III fracture (P<0.001). Type I and type II fractures were again showed to be associated with a better outcome (P=0.001 and 0.041 respectively).

As weight and BMI show a significant correlation (P<0.001 and correlation coefficient=0.743), only one item (weight) was included in the multivariate analysis. Multivariate linear regression analysis showed that displacement (P=0.005), weight (P=0.005) and type III fractures (P<0.001) were significant independent predictors of poor outcome at 6 weeks while at 20 weeks in addition to these factors, female gender and diabetes mellitus were also shown to be significant independent predictors of outcome.

*Risk Score*

**Figure 2:**
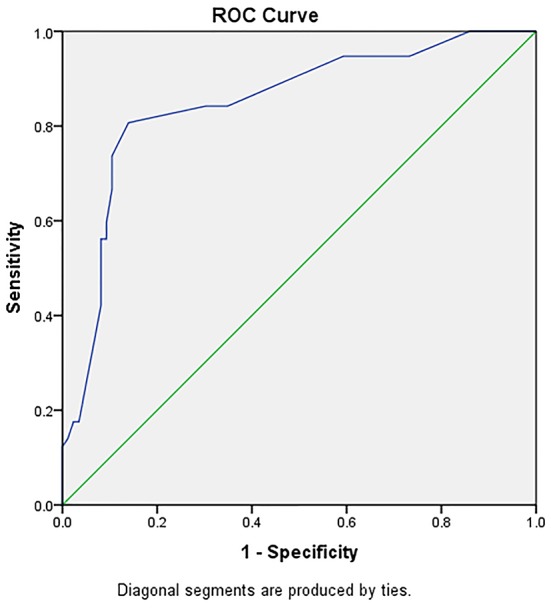
ROC curve showing the power of the score in predicting the outcome

We assigned scores to the predictive factors for the 20 weeks mid foot scale based on the natural logarithm of the coefficients in the multivariate analysis multiplied by 10 with rounding to the nearest integer and with consideration of the direction of their effect (table 2). We divided the patients into favourable and poor outcome at 20 weeks based on their mean MFS score (those with a score lower than the mean were considered as having a poor outcome). The ROC (Receiving Operating Characteristics) curve (figure 2) showed the score to have an AUC of 0.849 in predicting the outcome. The cut-off point was set at 9, that is, anyone with a score greater than 9 is at a higher risk of a poor outcome in 20 weeks.

**Table 2:** The Scoring System

**Variable**	**Score**
Gender (female)	8
DM	17
Displacement greater than 2 mm	19
Weight (>81 kg)	2
Type I fracture	-5
Type II fracture	0
Type III fracture	20

Internal validation of the model using the bootstrap method was performed and showed that there is consistent significant difference between the good outcome and poor outcome groups and thus our scoring system has good reliability in predicting the outcome in 20 weeks. 

## Discussion

Over the years, there have been many arguments for the best approach to treat proximal fifth metatarsal fractures with the current guidelines focusing on the type of fracture and degree of displacement. We have, however, demonstrated that other factors may play a role in the end outcome of the patients and while the general response to treatment was good among all patients after 20 weeks (as shown by the mean MFS score of 92.77 out of possible 100), there is a significant difference in outcome between individuals with certain risk factors and those without them.

Our study showed that while diabetes mellitus does not affect the outcome at 6 weeks, it plays a significant role in the outcome at 20 weeks. Fleischili et al. showed that the bone strength and stiffness of the metatarsal bones of the diabetic patients is similar to non-diabetic individuals who are 20 years older. While the evidence is not conclusive, it is believed that bone quality and healing in diabetic patients is inferior to non-diabetic individuals.^14^ This may explain why in our study we have observed a negative effect of DM on patients MFS score at 20 weeks.

We have observed a correlation between adverse outcome and female gender in our study. Hasselman et al. documented in a paper that foot fracture in females is associated with reduced bone density^15^ and this can play a role in delaying or complicating the healing process following fractures.

We also established that both BMI and weight are associated with negative effect on the outcome. We have, however, included weight in our scoring system as our study showed that height has no effect on the outcome and the observed effect of BMI on the outcome is in truth the effect of the weight, this effect is nonetheless limited and weight was only assigned a score of 2 in our scoring system.

Our study showed that fracture types have considerable effect on the outcome as well. Type I fractures leads to the best results while Type III fracture lead to poorer outcomes. While Type II fractures were shown to have an improved outcome in univariate analysis, this effect was not observed in multivariate analysis. Our findings are in line with the current evidence that suggest conservative and functional approaches to the treatment of type I fractures while type III fractures are best managed by surgical approaches. Type II fractures are controversial as both surgical and conservative approaches have been shown to be acceptable choices of treatment. Displacement of greater than 2 mm was also shown to be a negative predictive factor, which is again in line with the current evidence.^16^^-^^19^

Some have suggested that the fracture types; as suggested by Torg, are not sufficient in guiding the choice of decision; for example Logan et al. proposed a newer simplified classification that can guide the treatment and has higher interobserver agreement.^20^ While Lee et al. showed that plantar gap has a significant effect on the outcome of fifth metatarsal fractures; they used it to design a new classification and showed that its incorporation improves the decision supporting function of the classification.^21^^,^^22^ In our scoring system, while adhering to the original classification, we have identified other predictors that can affect the outcome; our study showed that individuals with scores of greater than 9 have poorer outcomes with conservative management and may be candidates for surgical intervention.

While we believe our scoring system to be a useful decision support tool in aiding the surgeon, we must caution that it should not replace sound clinical judgment on the part of the surgeon. The main limitation is that while our system has shown to be internally valid, it still needs to be externally validated in a further prospective cohort. 

## Conclusion

In short, our scoring system can help to identify patients who have a higher risk of failure using conservative treatment options; that is, a surgical approach to treatment may serve the patients with a score of more than 9 at best. 

## References

[1] Petrisor BA, Ekrol I, Court-Brown C (2006). The epidemiology of metatarsal fractures. Foot Ankle Int.

[2] Spector FC, Karlin JM, Scurran BL, Silvani SL (1984). Lesser metatarsal fractures. Incidence, management, and review. J Am Podiatry Assoc.

[3] Ekrol I, Court-Brown CM (2004). Fractures of the base of the 5th metatarsal. The Foot.

[4] Dameron TB Jr (1975). Fractures and anatomical variations of the proximal portion of the fifth metatarsal. J Bone Joint Surg Am.

[5] Lehman RC, Torg JS, Pavlov H, DeLee JC (1987). Fractures of the base of the fifth metatarsal distal to the tuberosity: a review. Foot Ankle.

[6] Richter M (2011). Fractures of the forefoot. Unfallchirurg.

[7] Yoho RM, Carrington S, Dix B, Vardaxis V (2012). The association of metatarsus adductus to the proximal fifth metatarsal Jones fracture. J Foot Ankle Surg.

[8] Zwitser EW, Breederveld RS (2010). Fractures of the fifth metatarsal; diagnosis and treatment. Injury.

[9] Strayer SM, Reece SG, Petrizzi MJ (1999). Fractures of the proximal fifth metatarsal. Am Fam Physician.

[10] Thevendran G, Deol RS, Calder JD (2013). Fifth metatarsal fractures in the athlete: evidence for management. Foot Ankle Clin.

[11] Zenios M, Kim WY, Sampath J, Muddu BN (2005). Functional treatment of acute metatarsal fractures: a prospective randomised comparison of management in a cast versus elasticated support bandage. Injury.

[12] Kitaoka HB, Alexander IJ, Adelaar RS, Nunley JA, Myerson MS, Sanders M (1994). Clinical rating systems for the ankle-hindfoot, midfoot, hallux, and lesser toes. Foot Ankle Int.

[13] Ibrahim T, Beiri A, Azzabi M, Best AJ, Taylor GJ, Menon DK (2007). Reliability and validity of the subjective component of the American Orthopaedic Foot and Ankle Society clinical rating scales. J Foot Ankle Surg.

[14] Fleischli JG, Laughlin TJ, Lavery LA, Shah B, Lanctot D, Agrawal CM (1998). The effects of diabetes mellitus on the material properties of human metatarsal bones. J Foot Ankle Surg.

[15] Hasselman CT, Vogt MT, Stone KL, Cauley JA, Conti SF (2003). Foot and ankle fractures in elderly white women. Incidence and risk factors. J Bone Joint Surg Am.

[16] Hatch RL, Alsobrook JA, Clugston JR (2007). Diagnosis and management of metatarsal fractures. Am Fam Physician.

[17] Khan W, Agarwal M, Warren-Smith C (2005). Management of fractures of the base of the fifth metatarsal distal to the tuberosity. The Foot.

[18] Polzer H, Polzer S, Mutschler W, Prall WC (2012). Acute fractures to the proximal fifth metatarsal bone: development of classification and treatment recommendations based on the current evidence. Injury.

[19] Smith TO, Clark A, Hing CB (2011). Interventions for treating proximal fifth metatarsal fractures in adults: a meta-analysis of the current evidence-base. Foot Ankle Surg.

[20] Logan AJ, Dabke H, Finlay D, Makwana N (2007). Fifth metatarsal base fractures: A simple classification. Foot and Ankle Surgery.

[21] Lee KT, Park YU, Young KW, Kim JS, Kim JB (2011). The plantar gap: another prognostic factor for fifth metatarsal stress fracture. Am J Sports Med.

[22] Lee KT, Park YU, Jegal H, Park JW, Choi JP, Kim JS (2013). Prognostic classification of fifth metatarsal stress fracture using plantar gap. Foot Ankle Int.

